# Clinical undergraduate training and assessment in primary health care: Experiences gained from Crete, Greece

**DOI:** 10.1186/1472-6920-5-13

**Published:** 2005-05-09

**Authors:** George Belos, Christos Lionis, Michael Fioretos, John Vlachonicolis, Anastas Philalithis

**Affiliations:** 1 Koropi Health Centre, Athens, Greece; 2 Health Planning Unit, School of Medicine, University of Crete, Heraklion, Greece; 3 Clinic of Social and Family Medicine, School of Medicine, University of Crete, Heraklion, Greece; 4 Laboratory of Biostatistics, School of Medicine, University of Crete, Heraklion, Greece

## Abstract

**Background:**

Primary Health Care (PHC) is increasingly being introduced into undergraduate medical education. In Greece, the Faculty of Medicine of the University of Crete was the first to introduce a 4-week long training in primary health care. This paper presents the experiences gained from the initial implementation of the teaching of practice-based primary care in rural Crete and reports on the assessment scale that was developed.

**Methods:**

284 students' case write-ups from the 6 primary care units (PCUs) where they were allocated for the period 1990 to 1994 were analysed. The demographic data of the students and patients and the number of home visits were studied. Content analysis of the students' write-ups was carried out, using an assessment scale consisting of 10 dichotomous variables, in order to quantify eight (8) primary qualitative criteria.

**Results:**

Internal reliability was estimated by the index KR20 = 0.67. Face and content validity was found to conform to the standards set for the course, while logistic linear regression analysis showed that the quality criteria could be used as an assessment scale.

The number of home visits carried out varied between the various different PCUs (p < 0.001) and more were reported in the write-ups that fulfilled criteria related to the biopsychosocial approach (p < 0.05). Nine quantitative criteria were fulfilled in more than 90% of case reports, but laboratory investigations were reported only in 69.0% of case reports. Statistically significant differences between the PCUs were observed in the fulfilment of criteria related to the community approach, patient assessment and information related to the patient's perception of the illness, but not to those related to aspects of clinical patient management. Differences in reporting laboratory investigations (p < 0.001) are explained by the lack of such facilities in some PCUs. Demographic characteristics of the patients or the students' do not affect the criteria.

**Conclusion:**

The primary health care course achieved the objectives of introducing students to comprehensive, community oriented care, although there was variation between the PCUs. The assessment scale that was developed to analyse the case-write ups of the students provided data that can be used to evaluate the course.

## Background

Over the past few decades, the necessity for community orientation of medical undergraduate training and for improving its integration with the health care system have been recognised by international and national bodies [1-3]. As a result, the majority of medical schools in the USA and Europe have embarked upon curricular initiatives to enhance practice-based primary care [4]. Although there is a variance in medical curricula across Europe, there is clear trend towards an increasing focus on primary health care (PHC), favouring a more generalist approach and setting educational objectives related to providing comprehensive care to ambulatory patients, taking account of the family circumstances of the patients, providing home care and integrating with the community.

The Faculty of Medicine of the University of Crete that opened in 1984 was the first medical school in Greece to include, since it was inaugurated, a four-week course in primary health care in the final year of the curriculum [5,6]. Students were allocated to one of 6 primary care units (PCUs) that collaborated with the Department of Social Medicine for this purpose. At that time, PHC was still in its early stages of implementation in the rural areas of Greece, with limited experience of a community-based approach and often lacking in facilities and staff. The collaboration has since developed into a network of PCUs with the medical faculty [7]. It was decided to carry out a retrospective study of the specific characteristics of this training during the earliest four years of the implementation of this course. This initial period was chosen because the essential features of PHC and of the biopsychosocial approach were still not incorporated into everyday practice, while the experiences gained during that phase influenced the content of the training in subsequent years. Drawing on an approach that was used in McGill University [8], an assessment scale was developed that measured qualitatively and quantitatively the content of the case write-ups of the students, for the purpose of evaluating the degree to which the training achieved the objectives of comprehensive PHC, based on the principles of the biopsychosocial model. The aim of this paper is to present the experiences gained from the initial implementation of the teaching of practice-based primary care in rural Crete and to report on the results of using the assessment scale that was developed for evaluating the course.

## Methods

### Setting

The six PCUs where the field work training in PHC took place: 5 on the Island of Crete and one on Santorini Island, from 1990 to 1994, starting when the first group of students reached the sixth year of their studies.

### Fieldwork training and students' assessment

The course syllabus included following the PCU's daily schedule of work under the supervision of the medical staff of the PCU, who acted as clinical tutors. Students were involved in the management of all ambulatory patients that attend, including acute and emergency cases, follow-up visits, preventive care activities, home visits and community based projects. They maintained a logbook of their daily tasks. The students were thus exposed to the knowledge, skills and attitudes required in PHC, including health promotion activities, addressing the living conditions of the population and becoming familiar with the effect of social circumstances on the health of the individual, the family and the community [5,6].

One of the tasks that the students had to carry out was to follow patients with a health condition that affected their social and psychological life. These patients were allocated to the students by the clinical tutors and the students visited the patients at home. Students were invited to choose one of these patients to present as a case study. This method gave them the opportunity to present cases that stimulated their personal interest, a practice in line with the principles of case-based learning and clinical competence based on medical records [9]. Students filled in a standardised case write-up that was prepared by the academic staff of the Department of Social Medicine and originated from the biopsychosocial model [5,6]. Irrespective of the primary care unit where the course took place, students chose cases of the same severity level. These cases were actually representative of the type and kind of cases served in the primary care settings in Greece and they correspond to the common diagnoses made in PHC in rural Greece [10].

The students' assessment was carried out by rating their performance during the course and the quality of their record keeping, and was performed on location by their clinical tutors. Also, the academic staff who led the course performed an oral final exam. A standardised, Visual Analogue Scale (VAS) – based instrument was used additionally for this purpose [6].

### Data Analysis

Ex post facto data from the case write-ups were analysed and reviewed. Two hundred eighty four students' standard case report write-ups (284) were collected. Three of these were excluded due to missing data. Most were of these write-ups were prepared by individual students, and 50 (17.8%) were prepared by student groups.

The criteria of quality assessment of the medical records were defined after content analysis of the requirements set by the Department of Social Medicine [5,6] and using standards set in the international literature [12-14]. In particular, 8 primary qualitative criteria were selected that addressed specific aspects of the training: (a) The community approach or relevance to PCU/ PHC, (b) Family record / structure or family pedigree, (c) Patient assessment / differential diagnosis or priority order, (d) Management strategy or plan for primary care, (e) Utility of PHC services / PCUs, (f) "Who" or person (information about the subject), (g) "What" or condition (information about the disease-illness-sickness of the subject), (h) "Where" or environment (information about the environment of the subject).

In order to quantify the quality of the records, ten variables, shown in table 7, were constructed, by slightly changing the order of the criteria and with the addition of the therapeutic approach and the availability of laboratory data. These variables were operationalised using a dichotomous approach, being assigned a value of 0 if absent and 1 if present and so made amenable to numerical analysis [15-17].

**Table 7:** Variables used in quantifying quality of records

Variable 1	Therapeutic approach of the issue patient, in the context of the given PCU
Variable 2	Laboratory data, in relation to the laboratory facilities of the given PCU
Variable 3	Community Approach: interrelation between patient's disease and primary care services
Variable 4	Family record: complete genogram or family pedigree, as well as record of existing dynamics within the family
Variable 5	Patient assessment: Prioritising of diagnostic problems and differential diagnosis (organisation of data), as well as the proposed steps and further measures needed for disease management within the primary care services
Variable 6	Management Strategy or Plan: Services suggested by the medical personnel of the PCU for the best possible management of the patient within the primary care services
Variable 7	Utility or Usefulness of primary care: Information related to the parts of the management plan that were actually implemented at the PCU and to the way this implementation offered positive or negative feedback to the PCU
9 and 10	All criteria covering the biopsychosocial point of view, as defined by Prof. Howard F. Stein [14], and in particular:
Variable 8	Information on "**Who**", i.e. about the subject-person (the way the patient perceives his/her disease or medical condition, and the effects on the patient's relationship with the other members of the family and the community) [3]
Variable 9	Information on "**What**", i.e. about the disease – illness – sickness perception of the subject, and the effects it has on the biological, functional and social level respectively
Variable 10	Information on "**Where**", i.e. data on the environment of the patient, and on how this environment affected the patient's medical condition or health problem [24-26]

Internal reliability and validity were assessed for this instrument, using 231 write-ups filled by individual students. They were considered satisfactory by split-half method of Kuder-Richardson-index, where KR20 = 0.67, on the ten quality criteria in table 7, since the concrete case write-ups were completed once during the course. Face and content validity were found to conform to the requirements set by the Department of Social Medicine. Structure validity and prognostic validity were proved using the linear regression through the origin method. Logistic linear regression analysis through the origin assessed the magnitude of the net effect of each independent variable on the sum-score (dependant variable: resulted as the sum of independent variables) [18,19].

Data from these case reports were classified according to content meaning and to the results of the survey, and were founded on the principles of content analysis [8]. From each case report, the following variables and demographic data were encoded: record date, PCU (1 to 6) where the course took place, gender of the participant, gender and age of the patient, type of the case (urgent, prescription visit, chronic disease), co-existing disease, number of student visits at the patient's home after first contact with the PCU for treatment and follow up (0 to 3 visits). The assessment was performed by two independent qualified generalists / reviewers in Koropi Health Centre in the Athens area. Their agreement was estimated, based on the following formula [20,21]:

, where:

x = the number of quality criteria common between the two (2) reviewers

ψ = the number of criteria used by reviewer A' (10, in total)

ω = the number of criteria used by reviewer B' (10, in total)

In this study, this equation formula is represented by a level of agreement β = 1.

Demographic data from the records were analysed with the usage of descriptive statistics. The level of statistic significance for quality parameters has been controlled with the x^2 ^test, and wherever it was appropriate, Yates correction or Fisher's exact correction was applied [[20,22], and [23]].

## Results

### Descriptive data

42.3% of case reports was processed by male students, 39.8% was processed by female students, while 17.7% was processed by student groups (Table 1). Patients' demographics are presented in table 2. Patients over 66 years old dominated the picture, representing 44.8% of male patients and 50.0% of female.

**Table 1:** Distribution of students per gender and PCU

**Primary Care Unit**	**Male Students N (%)**	**Female Students N (%)**	**Teamwork N (%)**	**Total**
PCU 1	19 (33.3)	23 (40.3)	15 (26.3)	57
PCU 2	17 (43.5)	13 (33.3)	9 (23.0)	39
PCU 3	32 (47.7)	26 (38.8)	9 (13.4)	67
PCU 4	20 (47.6)	14 (33.3)	8 (19.0)	42
PCU 5	28 (43.7)	30 (46.8)	6 (9.3)	64
PCU 6	3 (25.0)	6 (50.0)	3 (25.0)	12
**Total**	119 (42.3)	112 (39.8)	50 (17.7)	281

**Table 2:** Distribution of patients per gender and age group

**Age group**	**Male N (%)**	**Female N (%)**	**Total N (%)**
0–15 years	10 (8.0)	6 (3.8)	16 (5.7)
16–45 years	19 (15.2)	26 (16.7)	45 (16,0)
46–65 years	40 (32.0)	46 (29.5)	86 (30.6)
66+ years	56 (44.8)	78 (50.0)	134 (47.7)
**Total**	125	156	281

As for the number of home visits for health care and follow-up, 64.8% of the students performed a single visit and 31.0% performed two visits, while 2.8% of the students did not make any home visits at all (Table 3). The difference in the number of home visits between the PCUs reached statistical significance (p < 0.001). Whenever the write-ups fulfilled both the criteria of the family history and the biopsychosocial approach (variables 8, 9 and 10 in table 7), the numbers of home visits was larger, and vice versa. This difference reached statistical significance (p < 0. 05) (Table 4).

**Table 3:** Students' home visits per PCU

**Primary care unit**	**Number of home visits**			
	**0 N (%)**	**1 N (%)**	**2 N (%)**	**3 N (%)**

PCU 1	-(-)	48 (48.2)	9 (15.8)	- (-)
PCU 2	2 (5.1)	34 (87.2)	3 (7.7)	-(-)
PCU 3	3 (4.5)	37 (55.2)	25 (37.3)	2 (3.0)
PCU 4	2 (4.8)	19(45.2)	21 (50.0)	- (-)
PCU 5	- (-)	35 (54.7)	27 (42.2)	2 (3.1)
PCU 6	1 (8.3)	9 (75.0)	2 (16.7)	- (-)
**Total**	8(2.8)	182(64.8)	87(31.0)	4 (1.4)

**Table 4:** Co-existence of family structure and total biopsychosocial approach (N %) by average of home visits (HV).

**Primary care unit**	**Male N (%) / HV**	**Female N (%) / HV**	**Team N (%) / HV**
PCU 1	95/1.158	91/1.087	100/1.267
PCU 2	59/1.00	85/1.077	89/1.00
PCU 3	78/1.375	96/1.308	89/1.667
PCU 4	79/1.250	77/1.571	80/1.750
PCU 5	67/0.667	100/1.167	100/1.333
PCU 6	95/1.357	86/1.581	100/1.600

### Data on assessment

The complete data on the distribution of the assessment variables of the case write-ups by PCUs are presented in table 5. More than 95% of the case write-ups fulfilled criteria "1" (therapeutic approach), "7" (usefulness of primary care) and "10" ("Where" issues), while more than nine out of ten fulfilled criteria "4" (family history), "6" (management plan) and "9" ("What" issues). In all these cases, differences between the various PCUs were not significant or showed only weak statistical significance. More than 90% of the write-ups also reported on criteria "3" (community approach), "5" (patient assessment and differential diagnosis) and "8" ("Who" issues: the patient's perception of the illness), and in these cases the differences between PCUs were statistically significant. Only 69.0% of the write-ups fulfilled criterion "2" (appropriate laboratory investigation), the difference between PCUs being statistically significant (p < 0.001).

**Table 5:** Distribution of criteria for the assessment of the case write-ups per PCU

**Primary care unit**	**PCU 1 N (%)**	**PCU 2 N (%)**	**PCU 3 N (%)**	**PCU 4 N (%)**	**PCU 5 N (%)**	**PCU 6 N (%)**	**TOTAL**
1. Therapeutic Approach	98.2	92.3	98.5	95.2	98.4	100.0	97.2
2. Laboratory data (1)	66.7	38.5	62.7	100.0	70.3	100.0	69.0
3. Community Approach (2)	98.2	82.1	92.5	95.2	98.4	100.0	94.3
4. Family Record	98.2	84.6	91.0	92.9	93.8	100.0	92.9
5. Patient Assessment (3)	98.2	82.1	89.6	100.0	96.9	100.0	94.0
6. Management Strategy	94.7	92.3	88.1	90.5	98.4	100.0	93.2
7. Usefulness of primary care (4)	98.2	89.7	95.5	90.5	100.0	100.0	95.7
8. Who issues: Person (5)	100.0	97.4	93.9	100.0	82.8	100.0	94.3
9. What issues: disease – illness – sickness	96.5	92.3	95.5	95.2	85.9	91.7	92.9
10. Where issues: Environment (6)	100.0	100.0	95.5	97.6	90.6	100.0	96.4

(1) p < 0.001

(2) p < 0.01

(3) p < 0.01

(4) p = 0.06

(5) p < 0.001

(6) p = 0.06

The distribution of the criteria/variables of the case reports' assessment per gender and age group (Table 6) reveals that the individual demographic characteristics of the patients (age and gender) do not affect the quality of the training. Neither do the characteristics of the students (gender, individual or team work).

**Table 6:** Distribution of the criteria of the assessment of the case write-ups per gender and age group

**Age**	**0–15**		**16–45**		**46–65**		**66+**		**TOTAL**	
**Gender**	**Male N (%)**	**Female N (%)**	**Male N (%)**	**Female N (%)**	**Male N (%)**	**Female N (%)**	**Male N (%)**	**Female N (%)**	**Male N (%)**	**Female N (%)**

1. Therapeutic Approach	100.0	100.0	94.7	96.2	97.5	100.0	98.2	94.9	97.6	96.8
2. Laboratory data	70.0	83.8	73.7	65.4	55.0	84.5	69.6	65.4	65.6	71.8
3. Community Approach (1)	100.0	100.0	94.7	100.0	80.0	97.8	94.6	96.2	90.4	97.4
4. Family Record	90.0	100.0	89.5	92.3	87.5	95.7	91.1	94.9	90.4	94.9
5. Patient Assessment	90.0	100.0	89.5	96.2	90.0	95.7	96.4	93.6	92.8	94.9
6. Management Strategy	100.0	100.0	94.7	100.0	87.5	95.7	94.6	91.0	92.0	94.2
7. Usefulness of primary care	100.0	100.0	94.7	92.3	87.5	95.7	96.4	100.0	93.6	97.4
8. Who issues: Person	100.0	83.3	94.7	100.0	85.0	95.7	94.6	96.1	92.0	96.1
9. What issues: disease-illness-sickness	100.0	100.0	100.0	96.2	85.0	95.7	91.1	92.3	91.2	94.2
10. Where issues: Environment	100.0	100.0	94.9	100.0	97.5	97.8	94.6	94.9	96.0	96.8

(1) p = 0.06

After performing the linear logistic regression through the origin analysis (the no-intercept model), data relative to the estimation of predictive validity suggest all independent variables as predictors on the dependent one (overall sum-score) since all the above criteria-variables make a significant contribution to the cumulative score. According to these findings, the descending order of the independent variables-criteria is: 8 ("Who" issues), 4 (family record), 2 (laboratory data), 3 (community approach), 7 (usefulness of primary care), 1 (therapeutic approach), 6 (management strategy), and 5 (patient assessment) (Unstandardised Coefficients: R Square = 1.000, B = 1.000, Std. Error = .000, Standardised Coefficients: Beta = 0.126–0.129, level of significance p < 0.001).

## Discussion

This study was the first one of its kind ever to be performed in Greece. Although it is hampered by the lack of a control group, it is methodologically sound and provides useful information on the assessment of the whole training process. Teaching practice-based primary care requires medical students to understand the keys components of primary care, physicians to mobilise them and assessment tools for evaluating the undergraduate training.

Our study reports the first data available from the assessment of case write-ups. Case write-ups as an assessment method have an inherent weakness that could account for the fact that, despite its wide application, there are few studies that evaluate it as an assessment method. This weakness is that content analysis, method of choice for the assessment of the case write-ups, is inevitably subjective. To avoid this drawback, we carried out content analysis of our material by using a dichotomous (yes/no) approach of the qualitative variables. The level of agreement between the two reviewers about these quality criteria was found to be quite high (β = 1) which means full homogeneity and resemblance in the encoding between the two reviewers, underscoring the coexistence of objectiveness and content validity. Therefore, our results can be viewed only in the context of this methodological limitation. Further studies need to address the issue of concurrent validity of such an approach, complementing it with other methodological approaches that assess the educational process in a more detailed way, such as questionnaires with visual analogue scales or 5-point Likert scale and interviews with open-ended questions.

It is worth to note on the differences that were observed between the various PCUs. Thus, the number of home visits differed significantly among the PCUs (p < 0.001). This may be because time of our study some PCUs had developed home visits more than others. Further, the number of home visits proved to be an important factor for reporting on disease progress, as well as a means to evaluate factors related to the family and the social environment of the patient [12].

Regarding the criteria that were used to assess the case write-ups, it is interesting that those criteria that reflect the more clinical aspects of patient care were reported from almost all the PCUs, without significant differences. It is reasonable to assume that the clinical tutors in all the PCUs show the same interest in the treatment approach, the family history and the management plan, while they would encourage the students to report on the usefulness of PHC.

Reference to the patient assessment (the prioritisation of the elements referring to differential diagnosis) and to the community approach and its relation with PCU, was variably represented among the PCUs, reaching statistical significance (p < 0.01). The biopsychosocial approach ("who" issues) also received varying emphasis between the different PCUs (p < 0.001), although the overall rate was high. These differences may be attributed to a combination of factors: the actual contribution of the unit to the patient's management, the specific priorities adopted by each unit, the degree of familiarity of the clinical tutors with these aspects and the lack of interest in these parameters by the students.

Information obtained from the results of laboratory investigations was also found to differ significantly among the PCUs (p < 0.001). This can be explained by the fact that some of the units did not, at the time of the study, operate microbiology/biochemistry or radiology laboratories, and therefore it was not possible to carry out these investigations.

Since logistic linear regression through the origin analysis indicated that all of the proposed quality criteria function as predictors of the total assessment (in the aforementioned order), our findings are suggestive of a new total (trainee, trainer and the training site and program) assessment scale. This remains to be further investigated in the future as a useful methodological tool in ex post facto studies that are based on standardised medical records [24,25].

This study shows that the students' training achieved to a large extent the objectives that were set. However, achieving the objectives was affected by the orientation and the philosophy of the PCUs' personnel, and the attitude of the clinical tutors. On the other hand, individual demographic characteristics of the patients (age and gender), and the students (gender, individual or team work), as well as the disease / functional state of the patients, do not seem to affect the training.

Our study has several implications in undergraduate training in primary care. It is apparent that with the application of appropriate training courses, medical education can gradually shift from a model of illness and cure to a model of wellness and care [26,27]. However, emphasis should be given to support and/or select the PCUs that undertake to train students. The characteristics required to be fulfilled relate to the availability of basic medico-technological equipment as well as to the orientation towards primary care and the community. The fact that health care delivery in Greece is still highly fragmented and discontinuous makes it important to emphasise the holistic PHC approach and the biopsychosocial perspective. According to many authors, students are apt to immediately discern such characteristics [[9,12], and [13]].

At the same time, the assessment scale that we propose in this study proved to be a useful instrument in evaluating the entire training process (trainee, trainer, training site). Therefore, before participating in any training process, clinical tutors in PCUs must receive specific guidance on the quality criteria (table 7) used in the assessment of the students' case reports.

Almost ten years after this study was carried out, the Faculty of Medicine of the University of Crete remains to be the only one among seven medical schools in Greece to include primary care in its undergraduate curriculum. However, we know from our discussions with colleagues in other medical schools that they have recently started exploring the idea of introducing an elective course in PHC. In the meanwhile, the collaboration between the Department of Social Medicine and the Primary Care Units in Crete, which started as a means to train students in PHC, has developed into a network for health needs assessment [9] as well as for organisational and institutional development of PHC [28].

## Conclusion

This study demonstrates that the course in primary health care in the curriculum of the Medical Faculty of the University of Crete achieved to a large extent the objectives of introducing the students to a more biopsychosocial approach, although the degree of success varied between the PCUs where students were allocated. It also shows that the objectives of the training can be evaluated by using an analysis of the case write-ups that students prepare and present. This study also prompts us to re-examine the training that is taking place today, so as to ascertain whether the gradual establishment of a climate that favours PHC has contributed to improvements in the content of the course and to the fulfilment of the objectives that were defined during the early period of the course. Research in primary medical care education may benefit from the development of a consensus on assessment scales but further discussion and innovative methodology are required.

## Competing interests

The author(s) declare that they have no competing interests.

## Authors' contributions

GB collected the data, carried out the analysis and wrote the first draft of the manuscript. CL corrected the first daft, contributed to the analysis and interpretation of data, formed the layout of the manuscript and contributed to the manuscript's re-drafting. MF co-designed the contents of the training, initiated the collaboration with the PCUs and advised on the study design. The late JV carried out the statistical analysis and wrote the relevant sections. AP conceived the study design, co-designed the contents of the training and rewrote the manuscript. All the authors approved the final version of the manuscript.

## Pre-publication history

The pre-publication history for this paper can be accessed here:


